# Diet and Respiratory Health in Children from 11 Latin American Countries: Evidence from ISAAC Phase III

**DOI:** 10.1007/s00408-017-0044-z

**Published:** 2017-08-28

**Authors:** Alfonso Mario Cepeda, Sumaiyya Thawer, Robert J. Boyle, Sara Villalba, Rodolfo Jaller, Elmy Tapias, Ana María Segura, Rodrigo Villegas, Vanessa Garcia-Larsen, and the ISAAC Phase III Latin America Group

**Affiliations:** 1 Fundación Hospital Universitario Metropolitano de Barranquilla, Barranquilla, Colombia; 2 Population Health and Occupational Disease Group, NHLI Imperial College London, London, UK; 3 Department of Paediatrics, Faculty of Medicine, Imperial College London, London, UK; 4 School of Public Health, University of Chile, Santiago, Chile; 5 Department of International Health, Johns Hopkins Bloomberg School of Public Health, 615 N Wolfe, Suite E2546, Baltimore, MD 21205 USA

**Keywords:** Diet, ISAAC Phase III, Wheeze, Asthma, Latin America, Children, Fruits, Vegetables

## Abstract

**Background and Aim:**

The burden of childhood asthma and its risk factors is an important but neglected public health challenge in Latin America. We investigated the association between allergic symptoms and dietary intake in children from this region.

**Methods:**

As part of the International Study of Asthma and Allergies in Childhood (ISAAC) Phase III, questionnaire collected dietary intake was investigated in relation to risk of parental/child reported current wheeze (primary outcome) and rhino-conjunctivitis and eczema. Per-country adjusted logistic regressions were performed, and combined effect sizes were calculated with meta-analyses.

**Results:**

143,967 children from 11 countries had complete data. In children aged 6–7 years, current wheeze was negatively associated with higher fruit intake (adjusted odds ratio [aOR] 0.65; 95% CI 0.74, 0.97). Current rhino-conjunctivitis and eczema were statistically negatively associated with fruit intake (aOR 0.72; 95% CI 0.64, 0.82; and OR 0.64, 95% CI 0.56, 0.74, respectively). Vegetable intake was negatively associated with risk of symptoms in younger children, but these associations were attenuated in the 13–14 years old group. Fastfood/burger intake was positively associated with all three outcomes in the older children.

**Conclusion:**

A higher intake of fruits and vegetables was associated with a lower prevalence of allergic symptoms in Latin American children. Conversely, intake of fastfood was positively associated with a higher prevalence of wheeze in adolescents. Improved dietary habits in children might help reduce the epidemic of allergic symptoms in Latin America. Food interventions in asthmatic children are needed to evaluate the possible public health impact of a better diet on respiratory health.

**Electronic supplementary material:**

The online version of this article (doi:10.1007/s00408-017-0044-z) contains supplementary material, which is available to authorized users.

## Introduction

Asthma continues to represent a major public health burden worldwide. The Global Burden of Disease (GBD) reported that symptoms of asthma in low- and middle- income countries can be as high as the rates found in more developed countries [1]. In line with the large variations in economic and social development observed in Latin America, the prevalence of asthma is also diverse, with rates of current wheeze ranging between 4 and 30% in adolescents, and between 8 and 37% in younger children [1–3]. These rates are comparable to that of developed countries or those with higher economic wealth. Children with symptomatic asthma lose time off school and are less productive, with direct and indirect costs to themselves and society [4]. In children aged 5–14 years, asthma alone is in the top ten global ranking of disability-adjusted life years in children [5].

Despite the high rates of asthma in Latin America, studies examining the association between environmental risk factors (including diet) and allergic diseases have so far been scant [6]. Cross-sectional studies in Bolivia and Ecuador have shown a negative association between fruit intake and asthma symptoms, and evidence from two small case–control studies in Brazil showed conflicting results [7, 8]. In a sample of Colombian children, higher intake of fruits and vegetables was negatively associated with several allergic symptoms [9]. In young adults from Chile, dietary intake of flavonoids was associated with improved ventilatory function [10], but neither fruits, vegetables, or antioxidant nutrients were associated with subjective or objective measures of asthma [11]. The social inequalities and socio-demographic variations within and between countries of this region make interpretation of findings more difficult. The International Study on Asthma and Allergies in Children (ISAAC) has contributed to address the knowledge gap on dietary factors that could be related to asthma and allergic diseases [12].We aimed at investigating the association between dietary intake of foods with antioxidant, anti-inflammatory, and anti-allergic properties, with respiratory and allergic symptoms in children from across Latin America.

## Methods

### Sample

As part of ISAAC Phase III [13], eleven Latin American countries were included in this study (Argentina, Brazil, Bolivia, Chile, Colombia, Ecuador, Mexico, Panama, Peru, Venezuela, and Urugay) [14]. Participant countries collected information on dietary exposures and asthma-related symptoms following the ISAAC protocols.

### Outcome Assessment

The primary outcome for this analysis was current wheeze, which was defined as children (or they parents) answering yes to the question ‘Have you (Has your child) had wheezing or whistling in the chest in the past 12 months?’. Current rhino-conjunctivitis and current eczema were also analysed as secondary outcomes. The questions for current symptoms of rhino-conjunctivitis were ‘In the past 12 months, have you (has your child) had a problem with sneezing or a runny or blocked nose when you (he/she) did not have a cold or flu?’ and ‘In the past 12 months, has this nose problem been accompanied by itchy watery eyes?’. The questions for current symptoms of eczema were ‘Have you (Has your child) had this itchy rash at any time in the past 12 months?’ and ‘Has this itchy rash at any time affected any of the following places: the folds of the elbows, behind the knees, in front of the ankles, under the buttocks, or around the neck, ears or eyes?’. These questions were preceded by the question ‘Have you (Has your child) ever had an itchy rash coming and going for at least 6 months?’.

### Dietary Exposures

The questions on diet included in the Environmental Questionnaire (EQ) enquired about foods which had earlier been suggested to influence the risk of allergic diseases. Respondents were asked: ‘In the past 12 months, how often, on average, did you (did your child) eat or drink the following: fruit; vegetables (green and root); and fast food/burgers?’. Centres were encouraged to include local names to define foods if necessary. Frequency of food intake was categorised as follows: rarely or never (reference); once or twice per week; and ≥3 times per week (comparison). We investigated the association between allergic symptoms and these food groups for their possible antioxidant, anti- or pro-inflammatory effect on these diseases.

### Statistical Analyses

Data from centres of a same country were merged and results are presented per country. Distribution of general characteristics, potential confounders, and prevalence of outcomes are presented as percentages per country and for the total population. The three outcomes of interest were treated as binary variables (yes/no for having the disease). The association between each outcome and dietary exposures in each country was examined with multivariable logistic regressions, adjusting for potential confounders (physical activity (frequency of exercise per week), number of hours watching television (1, 2, or 3 per day), maternal education (primary, secondary, higher education), body mass index (BMI), and current maternal smoking (yes/no)). The adjusted odds ratios from the country-level analyses were meta-analysed to give an overall effect estimate. The I^2^ statistic was used to assess heterogeneity between countries.

### Ethical Approval

Each participant centre obtained their own funding and ethical approval prior to taking part in the study.

## Results

### Sample Characteristics and Prevalence of Allergic Symptoms

General characteristics of children aged 6–7 years are summarised in Table 1. A total of 53,635 children from seven countries had complete data on respiratory symptoms and dietary exposures. Over a third of the population (36.8%) reported watched television for at least 3 h a day, whilst only 24% of these children practised exercise three times per week. An overall 14% of mothers reported being current smokers, with the highest prevalence of smoking observed in Chile (35.7%) and the lowest (8.3%) in Brazil. The overall prevalence of current wheeze was 12.4% (95% confidence interval 12.1, 12.6).

**Table 1:** General characteristics of children participating in ISAAC Phase III in Latin America (age group 6–7 years)

Variable	Country							Total
	Brazil (*n* = 1070)	Chile (*n* = 6129)	Colombia (*n* = 12,679)	Mexico (*n* = 26,302)	Panama (*n* = 2943)	Venezuela (*n* = 3000)	Uruguay (*n* = 1512)	*N* = 53,635
Males (%)	49.4	48.8	46.9	49.7	48.5	47.1	47.8	48.7
Body mass index (BMI) (kg/(height)^2^ (Median, IQR difference)	16.5 (3.9)	17.4 (3.8)	16.5 (4.3)	16.5 (3.8)	16.5 (4.2)	17.2 (4.2)	16.3 (3.3)	16.6 (4.0)
≥3 daily hours watching TV (*n*, %)	246 (23.0)	2273 (37.1)	5219 (41.2)	9365 (35.6)	680 (23.1)	1686 (56.2)	379 (25.1)	19,758 (36.8)
Physical activity 3 times per week (*n*, %)	94 (8.8)	1829 (29.8)	3328 (26.2)	5770 (21.9)	1112 (37.8)	516 (17.2)	381 (25.2)	13,030 (24.3)
Current maternal smoking (*n*, %)	89 (8.3)	2190 (35.7)	988 (7.8)	2811 (10.7)	111 (3.8)	724 (24.1)	355 (23.5)	7268 (13.6)
Maternal secondary education (n, %)	341 (31.9)	2552 (41.6)	6312 (49.8)	13,246 (50.4)	978 (33.2)	1396 (46.5)	720 (47.6)	25,545 (47.6)
Respiratory outcomes (*n*, %)								
Current wheeze	183 (17.1)	987 (16.1)	1792 (14.1)	2063 (7.8)	659 (22.4)	599 (20.0)	341 (22.6)	6624 (12.4) (95% CI 12.1, 12.6)
Current rhino-conjunctivitis	178 (16.6)	760 (12.4)	1950 (15.4)	2998 (11.4)	335 (11.4)	611 (20.4)	102 (6.7)	6934 (12.9) (95% CI 12.6, 13.2)
Current eczema	62 (5.8)	823 (13.4)	1847 (14.6)	1523 (5.8)	411 (14.0)	371 (12.4)	119 (7.9)	5156 (9.5) (95% CI 9.3, 9.8)

The 13- to 14-year-old group was composed of 90,332 children from eleven countries (Table 2). Over half of them (57.1%) reported watching television for at least 3 h a day. Regular exercise (3 times per week) was practised by 27% of these children. Maternal current smoking showed large variations across countries, with a prevalence of 4% in Panama and 37% in Uruguay. In the majority of the countries, mothers completed secondary education. The overall prevalence of current wheeze was (13.3% (95% CI 13.1, 13.5) in the group of adolescents.

**Table 2:** General characteristics of children participating in ISAAC Phase III in Latin America (age group 13–14 years)

Variable	Country											Total
	Argentina	Bolivia	Brazil	Chile	Colombia	Ecuador	Mexico	Panama	Peru	Venezuela	Uruguay	N = 90,332
	(n = 6466)	(n = 3263)	(n = 9618)	(n = 10,689)	(n = 13,344)	(n = 3082)	(n = 29,747)	(n = 3184)	(n = 3022)	(n = 3000)	(n = 4917)	
Males (%)	51.4	47	48.1	47.9	46.8	49.2	49.2	49.4	64.1	51.9	48.4	49.2
Body mass index (BMI) (kg/(height)^2^ (Median, IQR difference)	19 (3.4)	20 (4.3)	19 (3.9)	20.4 (3.8)	18.6 (3.8)	20.3 (4.2)	20.6 (4.3)	18.6 (2.6)	19.7 (3.5)	20.5 (4.5)	19.8 (3.6)	19.8 (4.1)
≥3 daily hours watching TV (n, %)	3523 (54.5)	1712 (52.5)	5476 (56.9)	6809 (63.7)	9285 (69.6)	2094 (67.9)	15,339 (51.6)	739 (23.2)	1767 (58.5)	2109 (70.3)	2719 (55.3)	51,572 (57.1)
Physical activity 3 times per week (n, %)	1139 (17.6)	1002 (30.7)	2390 (24.8)	3581 (33.5)	3435 (25.7)	692 (22.5)	8282 (27.8)	1194 (37.5)	239 (7.9)	686 (22.9)	1700 (34.6)	24,340 (26.9)
Current maternal smoking (n, %)	2153 (33.3)	763 (23.4)	1761 (18.3)	3092 (28.9)	2084 (15.6)	424 (13.8)	5465 (18.4)	117 (3.7)	632 (20.9)	845 (28.2)	1815 (36.9)	19,151 (21.2)
Maternal secondary education (n, %)	2617 (40.5)	1258 (38.6)	2501 (26.0)	4952 (46.3)	6675 (50.0)	1550 (50.3)	11,153 (37.5)	1029 (32.3)	1023 (33.9)	1306 (43.5)	2592 (52.7)	36,656 (40.6)
Respiratory outcomes (*n*, %)												
Current wheeze	844 (13.1)	422 (12.9)	1974 (20.5)	1608 (15.0)	1584 (11.9)	479 (15.5)	2533 (8.5)	720 (22.6)	592 (19.6)	463 (15.4)	807 (16.4)	12,026 (13.3) (95% CI 13.1, 13.5)
Rhino-conjunctivitis	1209 (18.7)	713 (21.9)	1458 (15.2)	2153 (20.1)	3470 (26.0)	737 (23.9)	4460 (15.0)	364 (11.4)	560 (18.5)	746 (24.9)	512 (10.4)	16,382 (18.1) (95% CI 17.9, 18.4)
Eczema	454 (7.0)	664 (20.4)	413 (4.3)	1679 (15.7)	2119 (15.9)	409 (13.3)	1429 (4.8)	445 (14.0)	306 (10.1)	215 (7.2)	259 (5.3)	8392 (9.3) (95% CI 9.1, 9.5)

Only 62% of children aged 6–7 years and 61% of those aged 13–14 years reported eating fruits 3 or more times per week (Table S1, supplementary file) and less than half of the children in both age groups had an intake of vegetables 3 or more times per week.

### Association Between Respiratory Symptoms and Dietary Intake

#### Fruits

**Fig. 1:**
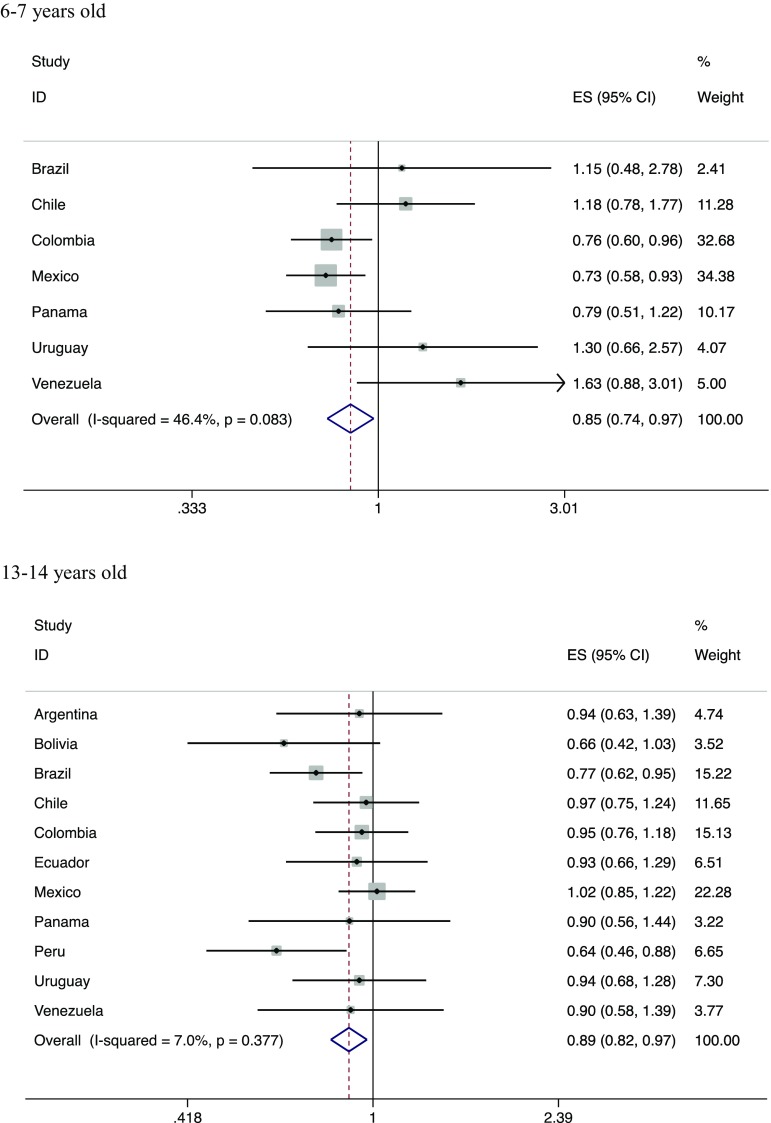
Meta-analyses of adjusted associations between current wheeze and fruit intake in children participating in ISAAC Phase III Latin America

Figure 1 shows the adjusted per-country associations between fruit intake and current wheeze. In children aged 6–7 years, there was a negative association between having current wheeze and eating fruits three or more times per week compared to less frequent consumption (adjusted overall OR 0.85; 95% CI 0.74; 0.97). The meta-analysis showed moderate heterogeneity (*I* ^2^ = 46.4%). In children aged 13–14 years, there was also a negative association between current wheeze and intake of fruits, although this was slightly attenuated (OR 0.89; 95% CI 0.82, 0.97). There was very little evidence of heterogeneity across countries (*I* ^2^ = 7.0%).

#### Vegetables

**Fig. 2:**
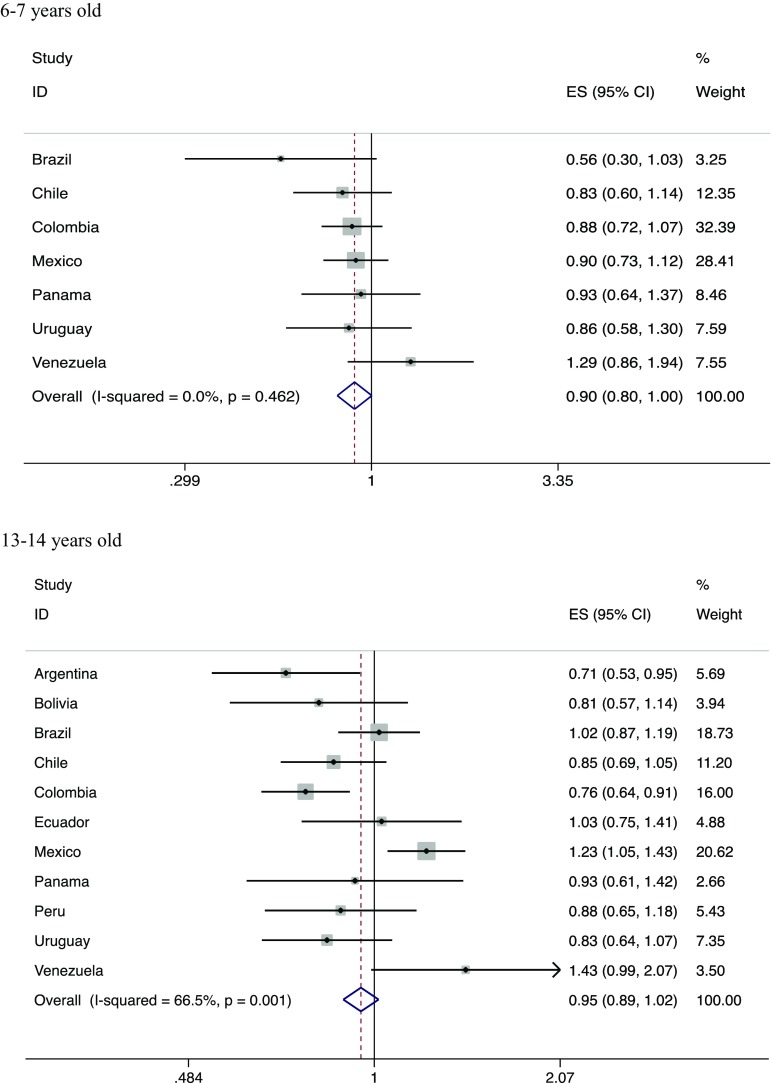
Meta-analyses of adjusted associations between current wheeze and vegetable intake in children participating in ISAAC Phase III Latin America

A higher intake of vegetables was negatively associated with current wheeze in children aged 6–7 years (Fig. 2; *I* ^2^ = 0%), whilst in older children there was no association, and there was moderate heterogeneity in the estimates across countries (Fig. 2; *I* ^2^ = 67%).

#### Fastfood

**Fig. 3:**
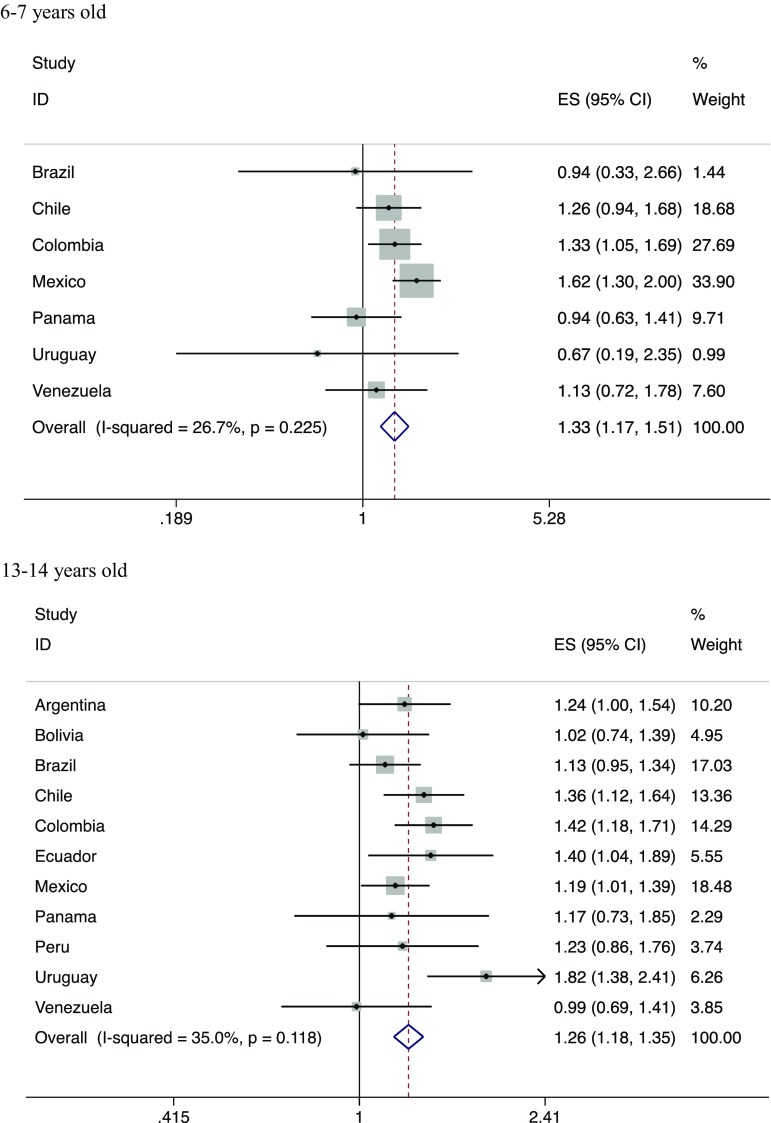
Meta-analyses of adjusted associations between current wheeze and fastfood intake in children participating in ISAAC Phase III Latin America

Current wheeze was positively associated with eating fastfood/burgers three or more times per week (Fig. 3). The positive associations were found in both age groups, with the effect size being slightly greater in younger children. Heterogeneity was small in both meta-analyses.

We also investigated the association between these food groups with secondary outcomes eczema and rhino-conjunctivitis (supplementary file). In the younger children, a more frequent intake of fruits was associated with a lower prevalence of eczema (OR 0.64; 95% CI 0.56, 0.74; Figure S1; and OR 0.72; 95% CI 0.64, 0.82, Figure S4), whilst the associations in the older children were less consistent (Figures S1 and S4). A more frequent intake of fastfood was positively associated with a higher prevalence of eczema and rhino-conjunctivitis in older children (Figures S3 and S6; ORs 1.32; 95% CI 1.22, 1.43, and OR 1.32; 95% CI 1.24, 1.40, respectively).

## Discussion

In this large multi-centre epidemiological study including 11 Latin American countries, we found that the overall prevalence of allergic symptoms in children aged 6–7 years fluctuated between 9.9% (current eczema) and 12.5% (current wheeze). We found a consistent trend across countries of a statistically significantly negative association between fruit, and vegetable, and allergic symptoms in the younger children. Such associations were attenuated in older children. We also found that frequent intake of fast foods or burgers was positively associated with a greater prevalence of allergic symptoms in adolescents and with wheeze in the younger children.

The meta-analyses on fruit intake and wheeze, eczema, and rhino-conjunctivitis showed very small heterogeneity in the group of 6- to 7-year-old children. The large sample size of the study, and the use of a standardised protocol to ascertain the prevalence of allergic symptoms and of frequency of food intake, might have contributed to the small heterogeneity. The fact that the effect sizes were fairly consistent across countries suggests that these associations might be biologically plausible. The heterogeneity and effect sizes were less consistent in the meta-analyses that examined dietary intake of vegetable and fastfood in adolescents, which might partly be explained by the increasing differentiation in the diet of older children.

Our findings also showed that three quarters of all the children studied had a sedentary lifestyle, reflected in the low frequency of physical exercise practised per week. Similarly, despite the strong public health promotion of daily intake of fruits and vegetables in children, we found that only 60% of the children eat these foods at least three times per week.

Diet is one of the key factors that can influence the susceptibility to allergic diseases [15] and can explain, at least in part, the current rates of asthma prevalence in Latin American countries. Latin America groups the Spanish and Portuguese-speaking countries south of the United States. In addition to the language, the population in this region has several common characteristics. The Latin American nations are largely composed of ‘Mestizos’ (those having Indigenous and Spanish/other European/African ascent) and indigenous people. The staple diet in most of these countries includes fresh cereals and vegetables (potatoes, maize, rice), although as with most growing economies, the effects of ‘Westernisation’ are increasingly departing the traditional diets towards consumption of fast food and other energy-dense and low-cost foods.

The ISAAC study provided for the first time standardised estimates of childhood asthma prevalence worldwide. ISAAC Phase Three produced internationally comparable estimates of direction and magnitude of change in symptoms of asthma, rhino-conjunctivitis, and atopic eczema [5]. The study also showed that in developing countries asthma and allergic disease were increasing.

We found that 75% of children had a fruit and vegetable intake below the five portions a day recommended by the WHO [16]. Worldwide variations in frequency of consumption of fruits and vegetables were reported for the first time in 2009 for adults from 52 countries, showing that lower income countries also have difficulties adhering to these recommendations [17]. The study showed figures for four countries from Latin America (Brazil, Ecuador, Paraguay, and Uruguay), all of which reported having at least 50% of adults eating less than 5 portions of fruits or vegetables per day. Our study is the first to demonstrate that the vast majority of Latin American children are not meeting these recommendations.

Despite the fact that asthma is an important public health burden in countries from this region [1], there have been few studies investigating the role that diet and other lifestyle-related risk factors might play in the risk of allergic diseases in Latin America. A negative association between fruit intake and childhood asthma has been suggested in low- and high-affluent countries [18]. A recent overview of high-quality systematic reviews on diet and asthma concluded that there is evidence to suggest that children who have a higher intake of fruits, or have diets which are rich in these foods, are less likely to suffer from asthma or allergic symptoms [19].

There is biological plausibility for the possible effect of fruits and vegetables on allergic symptoms. Oxidative stress plays a role in the inflammatory process that leads to the clinical expression of asthma and other allergic conditions such as eczema and rhino-conjunctivitis. Fruits and vegetables are rich in a number of antioxidant vitamins and minerals, as well as flavonoids whose antioxidant and anti-inflammatory properties have been suggested to reduce and modulate airway and allergic diseases. Experimental studies have demonstrated that flavonoids can inhibit the release of histamine and of several inflammatory cytokines including IL-4 and IL-13 [20], which are closely involved in the chain of events leading to the clinical manifestation of symptoms. Several well-designed randomised controlled trials (RCTs) have been carried out to test the demonstrability of such effects, but have so far shown little or no effect of the use of nutritional supplements on allergic outcomes [21].

A new concept of ‘indigenous microbiota’ which suggests that food consumed directly from the soil where it is harvested (rather than industrially produced) might also protect against the risk of asthma and allergies. It is known that fruits and vegetables are densely covered with microbiota (ectophytes), but the idea that these fresh products harbour a microbial world within (endophytes) has been only recently shown [22]. A low gut microbiota might be linked to poorer respiratory [23] and allergic [24] outcomes in children, and a rich microbiota is facilitated by freshly produced plant-based foods. Developed countries in Europe are now often able to market fruits and vegetables regardless of their seasonality, due to the high processing and artificial maturation these foods undergo. In Latin America, food markets are still common and widely accessed by the general population, often preferred to supermarkets (grocery shops) for their lower cost. Our findings could lend support to educational and intervention strategies to make fruits and vegetables more accessible to the population, like those investigated in other populations [25].

The introduction of a more ‘Westernised’ lifestyle has been increasingly suggested to play a role in the current burden of asthma in countries that are shifting towards a higher consumption of more energy-dense diet, which are characterised for their lower cost and high content of saturated fats. Our findings show that fast food intake in Latin America is very common in children, particularly in adolescents, of whom nearly a 20% reported eating these foods three or more times per week. The risk of current wheeze was positively associated with intake of fastfood in younger and older children, but this risk was higher in the adolescents. As consequence of the Westernisation of diet and of lifestyle in Latin America, the rates of cardio-metabolic diseases is increasing and the rates of obesity are a major public health challenge in the region [26]. The low cost of these foods and the high calorie content make fast food an appealing option for many families, in particular to those in a more vulnerable socio-economic position. The easy access that children have to processed and sugary food is also a major issue in many primary schools across the continent.

A major challenge to improve childhood global health in Latin America is to provide evidence from studies that can translate in public health interventions and in affordable and sustainable health care to the population [27]. Latin America is a region with large economic and social variations, and where public health resources are usually limited. Our results suggest that dietary intake of fruits might reduce the risk of allergic diseases in children, and that Westernisation of diet might contribute to the increase in the prevalence of allergic symptoms as children grow older. Timing appears to be an important factor, as the protective effect observed from higher intake of fruits and vegetables in younger children attenuates or disappears in older children. These results provide a foundation to inform public health policy.

## Electronic supplementary material

Below is the link to the electronic supplementary material.

Supplementary material 1 (DOCX 1217 kb)
