# Supplementary material for: Diet and Respiratory Health in Children from 11 Latin American Countries: Evidence from ISAAC Phase III
Source: Lung. 2017 Aug 28;195(6):683–92. doi: 10.1007/s00408-017-0044-z (PMC5674121; doi:10.1007/s00408-017-0044-z)
Supplement: Supplementary file 1 — Supplementary material 1 (DOCX 1217 kb) [file 408_2017_44_MOESM1_ESM.docx]

**Supplementary file ‘Diet and respiratory health in children from 11 Latin American countries– Evidence from ISAAC Phase III’, by AM Cepeda *et al*.**

**Table S1 Frequent dietary intake (≥3 times per week) of studied food groups in children participating in ISAAC Phase III Latin America**

|  | Children (n, %) | |
| --- | --- | --- |
| Food group | Age 6-7 years old | Age 13-14 years old |
| Fruits | 31,704 (62.1) | 52,912 (61.3) |
| Vegetables | 24,750 (49.0) | 39,631 (46.2) |
| Fast food/burgers | 5,112 (10.2) | 15,638 (18.2) |

**Figure S1 Meta-analyses of adjusted associations between eczema** **and fruit intake in children participating in ISAAC Phase III Latin America**

6-7 years old

13-14 years old

**Figure S2 Meta-analyses of adjusted associations between eczema and vegetable intake in children participating in ISAAC Phase III Latin America**

6-7 years old

13-14 years old

**Figure S3 Meta-analyses of adjusted associations between eczema and fastfood intake in children participating in ISAAC Phase III Latin America**

6-7 years old

13-14 years old

**Figure S4 Meta-analyses of adjusted associations between rhino-conjunctivitis** **and fruit intake in children participating in ISAAC Phase III Latin America**

6-7 years old

13-14 years old**Figure S5 Meta-analyses of adjusted associations between rhino-conjunctivitis and vegetable intake in children participating in ISAAC Phase III Latin America**

6-7 years old

13-14 years old

**Figure S6 Meta-analyses of adjusted associations between rhino-conjunctivitis and fast-food intake in children participating in ISAAC Phase III Latin America**

6-7 years old

13-14 years old
